# Sparse Representation of Sounds in the Unanesthetized Auditory Cortex

**DOI:** 10.1371/journal.pbio.0060016

**Published:** 2008-01-29

**Authors:** Tomáš Hromádka, Michael R DeWeese, Anthony M Zador

**Affiliations:** 1 Cold Spring Harbor Laboratory, Watson School of Biological Sciences, Cold Spring Harbor, New York, United States of America; 2 Department of Physics and Helen Wills Neuroscience Institute, University of California, Berkeley, California, United States of America; 3 Cold Spring Harbor Laboratory, Cold Spring Harbor, New York, United States of America; Porter Neuroscience Research Center, NIMH, United States of America

## Abstract

How do neuronal populations in the auditory cortex represent acoustic stimuli? Although sound-evoked neural responses in the anesthetized auditory cortex are mainly transient, recent experiments in the unanesthetized preparation have emphasized subpopulations with other response properties. To quantify the relative contributions of these different subpopulations in the awake preparation, we have estimated the representation of sounds across the neuronal population using a representative ensemble of stimuli. We used cell-attached recording with a glass electrode, a method for which single-unit isolation does not depend on neuronal activity, to quantify the fraction of neurons engaged by acoustic stimuli (tones, frequency modulated sweeps, white-noise bursts, and natural stimuli) in the primary auditory cortex of awake head-fixed rats. We find that the population response is sparse, with stimuli typically eliciting high firing rates (>20 spikes/second) in less than 5% of neurons at any instant. Some neurons had very low spontaneous firing rates (<0.01 spikes/second). At the other extreme, some neurons had driven rates in excess of 50 spikes/second. Interestingly, the overall population response was well described by a lognormal distribution, rather than the exponential distribution that is often reported. Our results represent, to our knowledge, the first quantitative evidence for sparse representations of sounds in the unanesthetized auditory cortex. Our results are compatible with a model in which most neurons are silent much of the time, and in which representations are composed of small dynamic subsets of highly active neurons.

## Author Summary

How do neuronal populations in the auditory cortex represent sounds? Although sound-evoked neural responses in the anesthetized auditory cortex are mainly transient, recent experiments in the unanesthetized preparation have emphasized subpopulations with other response properties. We quantified the relative contributions of these different subpopulations in the auditory cortex of awake head-fixed rats. We recorded neuronal activity using cell-attached recordings with a glass electrode—a method for which isolation of individual neurons does not depend on neuronal activity—while probing neurons with a representative ensemble of sounds. Our data therefore address the question: What is the typical response to a particular stimulus? We find that the population response is sparse, with sounds typically eliciting high activity in less than 5% of neurons at any instant. The overall population response was well described by a lognormal distribution, rather than the exponential distribution that is often reported. Our results represent, to our knowledge, the first quantitative evidence for sparse representations of sounds in the unanesthetized auditory cortex. These results are compatible with a model in which most neurons are silent much of the time, and in which representations are composed of small dynamic subsets of highly active neurons.

## Abstract 3

Patch clamp recordings in the auditory cortex of unanesthetized rats reveal that the population response to sounds is sparse and that most neurons are silent most of the time.

## Introduction

How does a population of cortical neurons encode a sensory stimulus such as a sound? At one extreme, the neural representation could be dense, engaging a large fraction of neurons, each with a broad receptive field. At the other extreme, the neural representation could be sparse, at any moment of time engaging only a small fraction of neurons, each highly selective with a narrow receptive field. Although a dense code under some conditions makes the most efficient use of the “representational bandwidth” [1] available in a neuronal population—why should a large fraction of neurons remain silent most of the time?—sparse models have recently gained support on both theoretical [2–4] and experimental [5–11] grounds. However, it is not at present clear which of these is a better model of sensory representations in the auditory cortex. In order to distinguish between these alternatives experimentally, we must know what fraction of neurons responds to a given stimulus.

The direct experimental approach to measuring the density of a cortical code would begin by simultaneously recording sound-evoked responses of all the neurons in the auditory cortex to an ensemble of stimuli; one could then simply count the number of spikes elicited by each stimulus. Unfortunately, currently available recording techniques do not permit such a direct approach. An alternative approach is to record the activity of a representative subset of neurons serially, and infer the population response from this sample. In this way, the population code could in principle be inferred by sequentially sampling a large population of single unit responses.

We have used cell-attached recording in the primary auditory cortex of unanesthetized rats to sample the population response to brief tones and other stimuli. Because we were interested in the population response, we presented a restricted ensemble of stimuli to each neuron, rather than optimizing the stimulus to drive each neuron to fire maximally [12,13]. Thus we could assess the fraction of neurons that responded for each stimulus we presented. The stimuli ranged from simple (tones, sweeps, white-noise bursts) to complex (natural sounds). Our data therefore address the question: What is the typical response across the entire neuronal population to a particular stimulus? Rather than: What is the optimal stimulus for a particular neuron? (see also [14]).

We find that the typical population response in unanesthetized auditory cortex is sparse. Consistent with previous findings in barrel cortex [10,15,16], some neurons had very low spontaneous firing rates (<0.01 spikes/second); at the other extreme, some neurons had driven rates in excess of 50 spikes/second. However, a given stimulus typically elicited a high firing rate (>20 spikes/second) in less than 5% of the population. Note that sparseness as used here refers only to the fraction of neurons active at a given instant; it is quite possible that each neuron might, under the appropriate conditions (e.g., when presented with an optimal stimulus), participate in a representation by firing at a high rate. Our results represent, to our knowledge, the first quantitative experimental support for the hypothesis that the representation of sounds in the auditory cortex of unanesthetized animals is sparse.

## Results

We recorded responses of neurons in the auditory cortex of head-fixed unanesthetized rats. Because our approach was to construct the population response one neuron at a time, we did not optimize the stimulus ensemble to conform to the response properties of each neuron, but instead probed many neurons with the same ensemble. In this way, we could reconstruct the overall population response.

We probed neurons with four different ensembles: tones (at three different intensities), sweeps, white-noise bursts, and natural sounds. These ensembles were selected because they span the range from spectrotemporally simple to complex.

**Figure 1:**
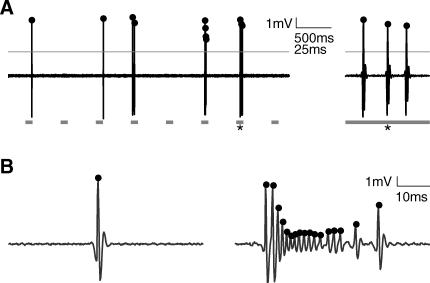
Cell-Attached Recordings in the Unanesthetized Auditory Cortex (A) Cell-attached recording allows for high-quality single-unit isolation. High-pass filtered voltage trace recorded in cell-attached mode in the auditory cortex of an unanesthetized rat. Spikes are easy to identify in the trace after thresholding (gray line). Spike times (dots) are assigned to peaks of suprathreshold segments. Gray squares indicate the positions of stimuli (pseudorandom sequence of 100-ms-long tones of different frequencies and attenuations). Note long time scale compared with other figures. The trace segment on the right shows the last 60 ms of the response to the 7^th^ stimulus (asterisk). (B) Spike shape can change significantly in a single neuron. Spike times (dots) are assigned to peaks of suprathreshold segments. The burst in the right panel preceded the single spike shown in the left panel by approximately 5 s. Both examples were tone-evoked, and occurred about 40 ms after stimulus termination. Although such dramatic changes are unusual, cell-attached recording minimizes the probability of both false positives and false negatives in spike detection.

Our goal was to record the responses to stimuli generated by a representative sampling of neurons in the auditory cortex. We therefore chose to record with a glass patch pipette in cell-attached mode, a method which is not explicitly biased toward active and responsive neurons, or neurons with large action potentials, and which provides excellent single unit isolation [10,17] (Figure 1). With cell-attached recording, single unit isolation depends on the physical contact between the glass electrode tip and the neuron. The selection bias of cell-attached recording is thus based on the neuron's “patchability,” rather than on the firing rate or responsiveness of the target neuron; only to the extent that patchability is correlated with functional characteristics such as firing rate or responsiveness would cell-attached recording (indirectly) bias the sampled population. In contrast, good single unit isolation with conventional extracellular (e.g., tungsten; [18]) electrodes requires a sufficient number of spikes; skilled practitioners typically search for neurons with sufficiently high firing rates and large spikes. Although it would be possible for a committed investigator to isolate neurons with a low spontaneous firing rate, for the purposes of this study cell-attached recording seemed a particularly suitable choice.

### Neuronal Responses Are Heterogeneous

Consistent with the earliest studies of unanesthetized auditory cortex [19–21], tones evoked a wide range of response patterns. Tones could elicit either an increase or a decrease in a neuron's firing rate over the background firing rate, or both; the change could be transient, delayed, or sustained; and the response pattern could be different for different tone frequencies in a single neuron.

**Figure 2:**
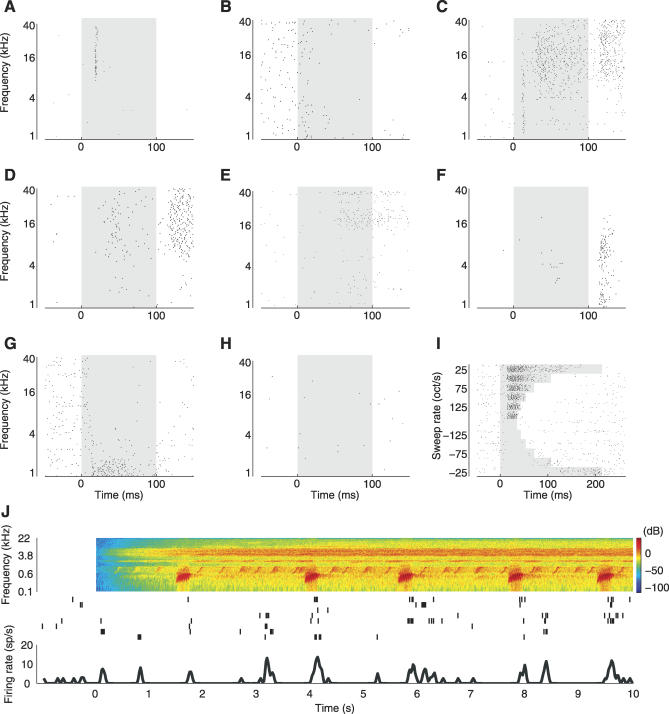
Sound-Evoked Responses in the Unanesthetized Auditory Cortex Are Heterogeneous (A–H) Tone-evoked responses in the auditory cortex of unanesthetized rats are heterogeneous. The panels show response dynamics of eight representative neurons to 60-dB tones. In each panel, dots represent individual spikes, the gray shaded region indicates the tone duration (100 ms). (A) transient onset response; (B) suppressive response; (C) transient onset response followed by sustained excitatory response followed by off response; (D) late onset response followed by strong off response; (E) late onset response; (F) off response; (G) sustained response combined with suppressive response; (H) non-responsive cell. See also Figures S4–S8 for more examples. (I) Example of single neuron responses to 54-dB sweeps. Dots represent individual spikes, the gray shaded regions indicate the stimulus duration. (J) Example of single neuron responses to natural sound (Knudsen's frog). Spectrogram of stimulus is shown at top (red indicated highest intensities and blue indicates lowest intensities), and individual trials are plotted in the middle (ticks represent spikes). Firing rate curve in the bottom of the panel was computed by first summing the spikes in 20-ms bins and then convolving the resulting peristimulus time histogram (PSTH) with a Gaussian (σ = 20 ms). Note the long time scale compared to the other panels.

Figure 2 shows some examples of the range of response types we observed. In one neuron (Figure 2A), tones elicited a transient, short latency response of the sort commonly observed in the barbiturate-anesthetized auditory cortex. In a second neuron (Figure 2B), tones elicited a suppression of background activity. In a third neuron (Figure 2C), higher frequency tones (∼8–40 kilohertz [kHz]) elicited vigorous sustained firing; interestingly, lower-frequency tones elicited transient responses in the same neuron, emphasizing that the distinction between “transient” and “sustained” applies to responses, not neurons. Other more complex response patterns were also observed (Figure 2D–2G.) Finally, half of the neurons tested (50%, see below) showed no change in firing rate for any stimulus presented (Figure 2H). Because a given neuron could show very different response patterns to stimuli of different frequencies (e.g., Figure 2C), we could not find a simple and objective scheme for organizing neurons into a small number of distinct classes, such as “transient,” “sustained,” “off,” etc. The neurons shown in Figure 2 are a subset; the complete set of responses from the entire dataset is shown in the Figures S4–S8.

### Population Response Is Lognormally Distributed

**Figure 3:**
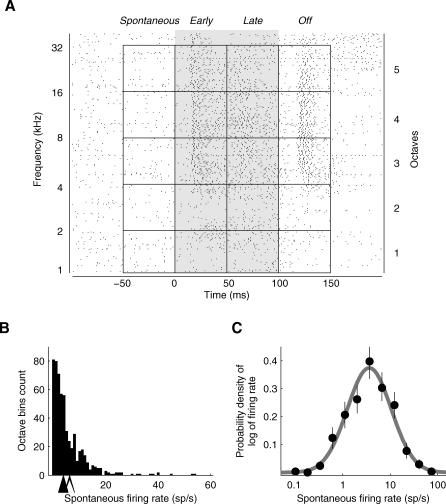
The Distribution of Firing Rates Follows a Lognormal Distribution (A) Cells were characterized by their activity during each of the response epochs: *spontaneous*, *early*, *late*, and *off*, each 50 ms long. *Spontaneous* epochs cover spontaneous activity before the stimulus, *early* and *late* epochs cover first and second half of the stimulus duration (100 ms), respectively, and *off* epochs cover 50-ms period after stimulus termination. In frequency space, individual trials were grouped into one-octave-wide bins, and averaged to provide a firing rate value for each octave bin. This figure shows a spike raster plot for an example neuron (with a sustained excitatory response), where each row represents a single trial, and each dot marks the occurrence of a spike. Shown are responses to 1–40 kHz tones (60 dB SPL, left ordinate.) Individual trials were grouped into five spontaneous, and 15 evoked response bins (right ordinate.) Note that the top quarter of an octave is not included in any of the bins. (B and C) Firing rates of most neurons were low and followed a lognormal distribution. (B) Frequency histogram of nonzero spontaneous firing rates in individual octave bins (*n* = 567 octave bins, from 145 neurons). Each neuron contributed a maximum of four or five data points (because each neuron had four or five octave bins per epoch). The filled arrow shows the position of the median spontaneous firing rate, and the open arrow shows the position of the mean spontaneous firing rate. (C) The distribution of spontaneous firing rates (dots) was fit with a lognormal distribution (gray line), the mean and variance of which were given by the mean and variance of the original firing rate distribution (see Materials and Methods). The lognormal distribution appears as a normal distribution on a (semi-) logarithmic scale. The error bars show 95% confidence intervals determined by bootstrapping.

We first analyzed the basic population response elicited by tones, beginning with the response to tones presented at 50 or 60 decibels (dB SPL). We divided the tone-evoked response into four 50-millisecond (ms) long “epochs”: *spontaneous*, *early*, *late*, and *off* (Figure 3A, also see Materials and Methods). To ensure a sufficient number of trials for assessing the statistical significance of putative changes in firing rate over background, we grouped responses across nearby frequencies (one-octave-wide bins; four- or five-octave bins for each response epoch). Control analyses using narrower (half-octave) bins gave similar results (see Materials and Methods), as expected from the relatively broad frequency tuning of neurons in the rat primary auditory cortex [22,23]; see also [24].

Both spontaneous and evoked firing rates were typically low (see Figure 3 and Table 1). The median spontaneous firing rate across the population was 2.8 spikes/second (sp/s). The mean was somewhat higher (4.9 sp/s) because it was dominated by a relatively small set of neurons—possibly interneurons (see Very Responsive Neurons May Be Narrow-Spiking Interneurons, below)—with high spontaneous rates.

**Table 1:**
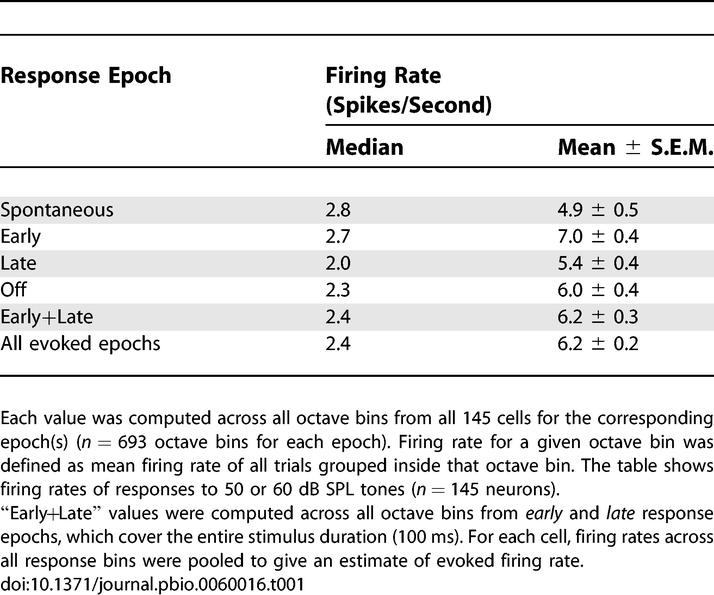
Firing Rates for Different Response Epochs Were Typically Low.

Evoked firing rates showed the same pattern: a low median (2.0–2.7 sp/s) and a somewhat higher mean (5.4–7.0 sp/s). The higher mean rates reflect the fact that in some neurons, some frequencies evoked vigorous firing (see Figure 2C for an example). However, such well-driven responses were the exception rather than the rule; as quantified below, most neurons did not respond vigorously to any of the tones presented. Note that for a neuron to contribute on average at least one spike to the population representation of a sound in a 50-ms window, its evoked firing rate must exceed 20 sp/s.

To assess whether the low firing rates resulted from some intrinsic defect of the spike-generating mechanism, perhaps introduced by the cell-attached recording method, we extracted the shortest interspike interval (ISI) for each neuron. In most neurons, the shortest ISI was less than 10 ms (median shortest ISI = 4 ms, *n* = 145 cells). Thus, the low firing rates do not appear to arise from an intrinsic inability of neurons to fire rapidly, but instead presumably arise from differences in the synaptic drive received by different neurons.

**Figure 4:**
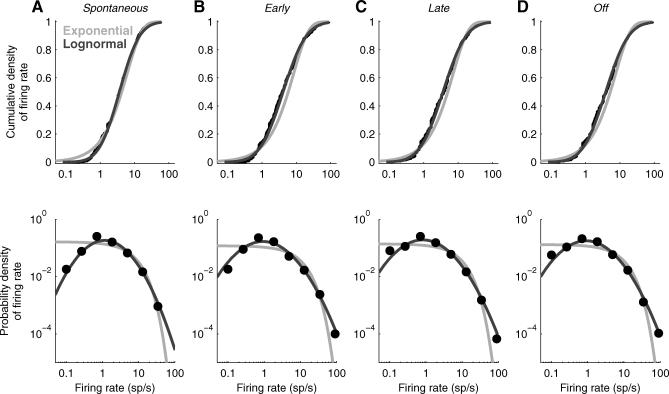
A Lognormal Distribution Provides a Better Fit to the Data than an Exponential Distribution (A) The cumulative density (top) and probability density (bottom) functions of the data (black points) are better fit by a lognormal distribution (dark gray line) than an exponential distribution (light gray line) for *spontaneous* firing rates. The mean and standard deviation of the lognormal fit were given by the mean and standard deviation of the distribution of (natural) logarithms of the firing rates. The mean of the exponential fit was given by the mean of the firing rate distribution. (B, C, and D) Same format as in (A) for *early, late,* and *off* epochs, respectively.

The distribution of spontaneous firing rates across the population was remarkably well fit with a lognormal distribution—that is, the logarithm of the firing rates was well fit with a Gaussian distribution (Figure 3B and 3C). Because the lognormal distribution has a “heavy tail,” most spikes were generated by just a few neurons: About 16% of neurons—the subset of 23 neurons firing at higher than 9.5 sp/s—accounted for 50% of all spikes. The lognormal distribution fit better than the exponential distribution, particularly at low firing rates (Figure 4); because we were using cell-attached recording, we were confident that we were not undersampling the low-firing end of the distribution and that therefore this improved fit was real. Although lognormal distributions have widely been used to describe the ISI distributions from a single neuron, population responses are usually reported to be exponentially distributed [6,25,26]; this is, to our knowledge, the first report that firing rates across a population of neurons are lognormally distributed.

### The Population Response Is Sparse

**Figure 5:**
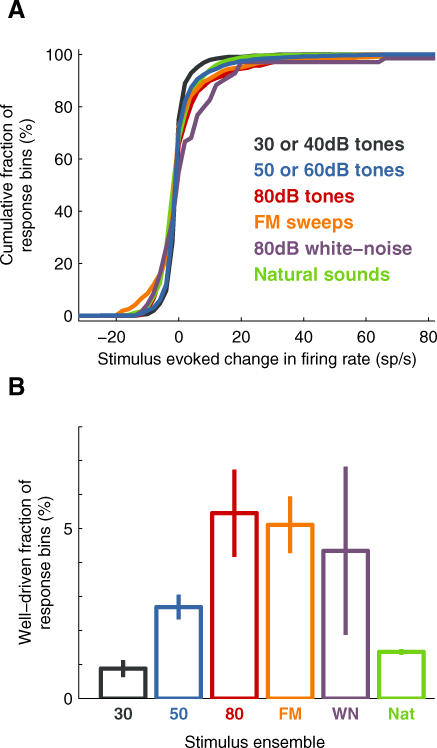
Only a Small Fraction of the Population Showed a Well-Driven Stimulus-Evoked Response at Any Instant (A) Cumulative fraction of stimulus-evoked changes in firing rate for various stimuli. Different colors correspond to different stimuli: black: 30 or 40 dB tones (*n* = 91 neurons, 1,365 response bins); blue: 50 or 60 dB tones (*n* =145 neurons, 2,079 response bins); red: 80 dB tones (*n* = 22 neurons, 330 bins); orange: FM sweeps (*n* = 22 neurons, 704 bins); purple: 80 dB white-noise bursts (*n* = 23 neurons, 69 bins); and green: natural sounds (*n* = 27 neurons, 18,900 bins). (B) Fractions of response bins showing well driven (>20 sp/s) stimulus-evoked change in firing rate were low for all stimulus ensembles used. Error bars show standard error determined by bootstrapping.

What is the typical response across the entire neuronal population to a particular stimulus? Figure 5 shows the cumulative distribution of firing rate changes (with respect to baseline) for each of the stimuli tested. To simplify the interpretation of these cumulative distributions, we defined an arbitrary threshold of 20 sp/s, beyond which we labeled the response as “well-driven;” Figure 5B shows the fraction of neuronal population exceeding this threshold for each ensemble. The choice of 20 sp/s, which corresponds to only a single extra spike in the 50-ms response bin, we consider was quite conservative; for example, other authors have chosen a higher (arbitrary) value of 50 sp/s as the threshold for the “high-firing” regime [27].

The typical stimulus-evoked population response was sparse for all stimulus ensembles tested: tones, sweeps, white-noise bursts, and natural sounds. Only a small fraction—less than 5%—of the population showed a well driven (>20 sp/s) response. That is, only a few percent of the neuronal population was likely to fire one or more “extra” action potentials (above baseline-firing rate) within any 50-ms window, regardless of the stimulus (see also Figure S3).

Such a sparse response might seem incompatible with evidence from other recording technologies, such as multiunit recordings (or fMRI), indicating that tones and other stimuli can indeed elicit substantial increases in population activity. However, these lines of evidence can be reconciled: even though only a small fraction of neurons was highly active at any instant, the activity in this small fraction could lead to as much as a 50% increase in the mean (as opposed to the median) firing rate (e.g., 4.9 versus 7.0 sp/s during the early tone epoch; see Table 1). Thus the presentation of a stimulus caused only a barely discernible change in the activity of most of the population; but an appreciable number of extra spikes were concentrated in a small fraction of neurons.

Although the distributions of firing rate changes were similar across stimulus ensembles, we did observe minor differences in the mean driven rate among ensembles. As expected, louder tones elicited a greater population response than quiet tones. Perhaps surprisingly, simple spectrotemporally rich stimuli (sweeps) elicited a somewhat greater response than did complex stimuli (natural sounds). Nevertheless, these differences were relatively small, and qualitatively the ensembles elicited similarly sparse population responses.

The analysis so far has focused on the fraction of well-driven neurons. What fraction of neurons showed any detectable stimulus-locked change at all, beyond those predicted by chance fluctuations around the baseline? Note that the definition we use here for responsiveness is quite inclusive: Even if a tone elicited only a 1 sp/s increase over the baseline-firing rate, this response might still be deemed responsive if the spontaneous rate was sufficiently low for us to detect a change.

**Figure 6:**
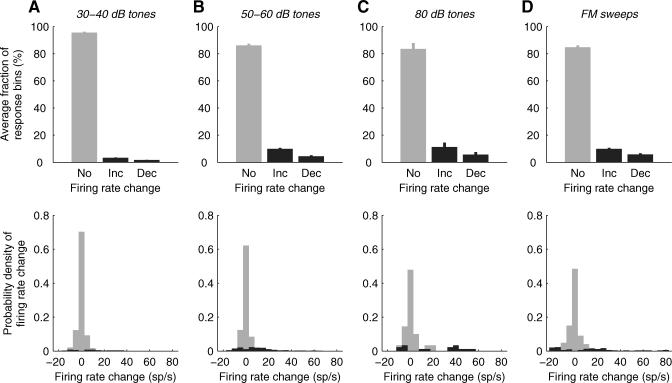
Only a Small Fraction of the Neuronal Population Participated in the Response to an Acoustic Stimulus at Any Instant (A) The top panel shows the fractions of response bins per octave displaying no change (No), a significant (*p*<0.01) increase (Inc), or a significant decrease (Dec) in the firing rate evoked by 30–40 dB tones (see Materials and Methods for details). Error bars show standard errors of the fractions of response bins. The bottom panel shows frequency histograms of firing rate changes in the response bins from the top panel. The gray histogram shows firing rate changes for response bins from the “No” bar from the top panel, and the black histogram shows firing rate changes for response bins from the “Inc” and “Dec” bars from the top panel. Both panels in (A) include data from 930 response bins (*n* = 62 neurons). (B, C, and D) Same format as in (A) for 50–60 dB tones (B), 80 dB tones (C), and FM sweeps (D). Panel (B) includes data from 1,404 response bins (*n* = 100 neurons), panel (C) includes data from 90 response bins (*n* = 6 neurons), and panel (D) includes data from 544 response bins (*n* = 17 neurons).

The majority of neurons showed no discernible response to any stimulus during any given response epoch. Figure 6 shows the evoked population response to each stimulus ensemble (with the exception of natural stimuli and white-noise bursts, for which not enough repetitions were presented; see Materials and Methods). During each 50-ms response epoch only about 10% of neurons showed any significant stimulus-locked increase in firing rate (Figure 6 top, *Inc*), and a smaller fraction showed a significant stimulus-locked decrease (Figure 6 top, *Dec*). Thus, not only was the fraction of well-driven neurons low, the fraction of neurons driven at all was also low.

Half of the cells (50%) did not show any significant change (increase or decrease) in firing rate during any response epoch, to any stimulus; an example of such an unresponsive neuron was shown in Figure 2H. At the other extreme, a few broadly tuned cells showed significant changes in firing rate in all (four or five) octave bins (i.e., across the whole frequency space tested) for at least one of the response periods.

It might appear that the sparseness we report is incompatible with the broad frequency tuning of rat auditory cortical neurons. However, we found that sparseness was not achieved through narrow frequency tuning. Instead, it arose through a combination of factors. First, 50% of the neural population failed to respond to any of the simple stimuli we presented. Second, responses were often brief; in many neurons, the change in firing rate was limited to just one of the three response epochs. Thus, sparseness of the response in time contributed to the overall sparseness of the population response. Finally, even when changes occurred they were typically small; the increase in firing rate exceeded 20 sp/s in only about a quarter of the statistically significant responses. As a result, only a small fraction of neurons responded vigorously to any tone even though frequency tuning was broad.

The form of sparseness we report has sometimes been termed “population sparseness,” to distinguish it from “lifetime sparseness” [2,28]. Lifetime sparseness refers to the selectivity of a single neuron probed with different stimuli and can be assessed for a single neuron during a single unit experiment. Population sparseness refers to the response of the population to a given stimulus. Responses in visual cortex have been reported to show population sparseness [29], but population sparseness has not previously been assessed in auditory cortex.

### Neither Spatial nor Laminar Position Predicts Response Pattern

The heterogeneity of response patterns to simple tones led us to wonder whether neurons with similar properties might be clustered into nearby regions of the cortex; for example, neurons with predominantly transient responses might be found in one region, and sustained neurons might be found in another. In some cases, therefore, we recorded from multiple cells in a single electrode penetration. Since the recording electrodes were aligned approximately perpendicular to the cortical surface, the cells recorded in a single electrode penetration likely belonged to the same or neighboring cortical column.

**Figure 7:**
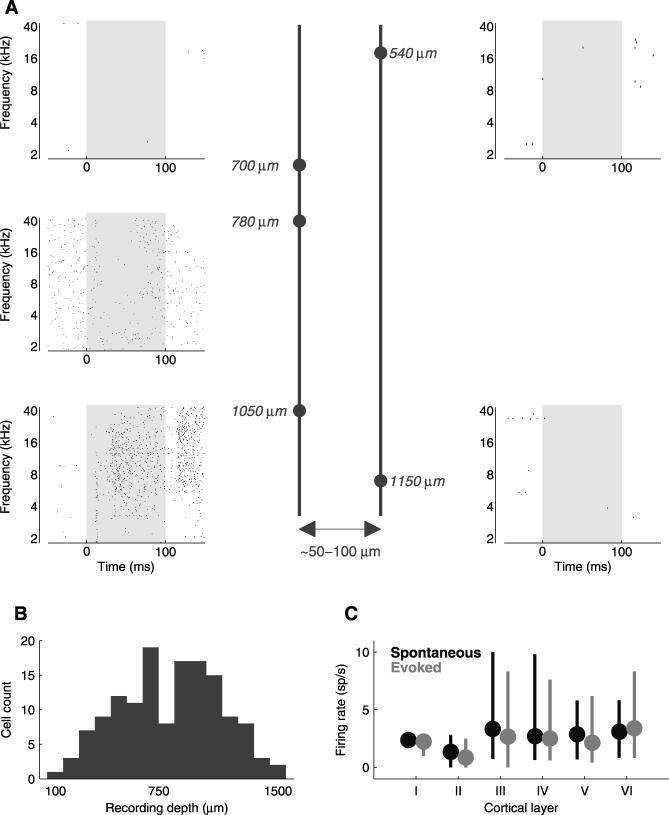
Neither Spatial nor Laminar Position Predicts Response Pattern (A) Neurons recorded in a single penetration can show very different tone-evoked responses. Response rasters are shown from neurons recorded in two penetrations (left and right columns); the penetration depicted in the right column was about 50–100 μm ventrocaudal from the penetration depicted on the left. Dots represent individual spikes and gray shaded regions indicate the tone duration (60 dB, 100 ms). Depths of recordings were measured perpendicular to the cortical surface, as given by micromanipulator readings and as such are approximate. The cell shown in the lower left corner is the same as that in Figure 2B. (B) Frequency histogram of recording depths shows that all depths were represented in our sample. All depths (*n* = 141 cells) were given by the micromanipulator reading, and as such are only approximate. For four cells the depth information was not available, and they are not included in the figure. (C) Firing rates were similar across cortical layers. Neurons (*n* = 141) were segregated into six groups corresponding to cortical layers (see text for details). Spontaneous firing rates were computed for each response bin. Evoked firing rates were computed in all evoked response bins. Circles show the positions of medians, error bars extend from the lower quartile (25^th^ percentile) to the upper quartile (75^th^ percentile).

We did not detect any clustering of response patterns; highly responsive cells were often very near unresponsive cells. Figure 7A shows an example with five neurons recorded over two penetrations (three in one penetration, and two more in a penetration approximately 50–100 μm ventro-caudal from the first penetration). In the first penetration, one neuron was unresponsive, one showed suppression over a wide range of frequencies, and the third showed enhanced firing over an even wider range of frequencies. In the second penetration, both neurons were unresponsive. The fact that unresponsive neurons were often mixed closely with responsive neurons indicates that unresponsiveness need not indicate gross cortical damage (see also [21]) or recording from a region of cortex that was unresponsive to the stimuli we were presenting, but that instead neurons with different selectivity are commingled.

We also wondered whether firing rate was correlated with cortical layer. We segregated neurons (*n* = 141) recorded at different cortical depths (Figure 7B, depths were estimated using the micromanipulator readings and as such were only approximate; see Materials and Methods) into six groups corresponding to the cortical layers [30] (Figure 7C). We compared the firing rates using multiple comparisons based on Kruskal-Wallis test and found that the spontaneous and mean evoked firing rates were not significantly different, with the exception of layer II, which displayed firing rates significantly lower (*p* < 0.01) than the other cortical layers (layer I contained only one neuron and was not included in the comparisons). Thus, cortical layer does not seem to account for the diversity of response properties we observed.

### Very Responsive Neurons May Be Narrow-Spiking Interneurons

Because we could record from only a relatively small number of neurons in a single penetration, we cannot rule out the possibility that more thorough sampling of all the nearby neurons in a region might reveal subtler forms of spatial or laminar organization that escaped our detection. Alternatively or additionally, responsiveness might be correlated with single neuron properties such as type, morphology, and molecular expression pattern. Although in this study we did not recover neurons for histological analysis and so could not assess whether there was a correlation with morphology or molecular expression pattern, we did attempt to correlate responsiveness with cell type.

Cortical neurons can be grouped into two broad classes: excitatory neurons that release glutamate at their synapses; and inhibitory interneurons, which release gamma-aminobutyric acid (GABA). Most cortical neurons are excitatory. GABAergic neurons can have diverse morphological, physiological, or molecular characteristics [31]. Excitatory and inhibitory neurons can also be distinguished based on a variety of physiological parameters [32,33]. In particular, the firing rate of some inhibitory interneurons—the so-called fast-spiking subtype—is higher when stimulated by current injection. Spike width and shape have been used in previous studies to assign spikes recorded extracellularly in vivo to putative excitatory and inhibitory neurons in hippocampus [34], and cortex [35]. We therefore asked whether spike shape might predict response patterns in our sample.

Based on previous studies (for example, [32,33]), we expected that fast-spiking interneurons would likely have narrow and symmetric spikes. For each cell we therefore computed the spike width, and also the “spike amplitude index” as a measure of spike symmetry (see Materials and Methods). For our population of cells the spike widths ranged from 0.4 ms to 1.9 ms, with a median value of 0.9 ms. We defined the spike amplitude index as the absolute value of the spike peak-to-valley-ratio. A spike amplitude index of unity indicates a perfectly symmetrical spike, whereas a value greater than unity indicates a tall spike, and a smaller value indicates a spike with a deep valley; a fast-spiking interneuron would be expected to have a low spike amplitude index. Spike amplitude indices ranged from 0.8 to 34.3, with a median value of 2.0.

**Figure 8:**
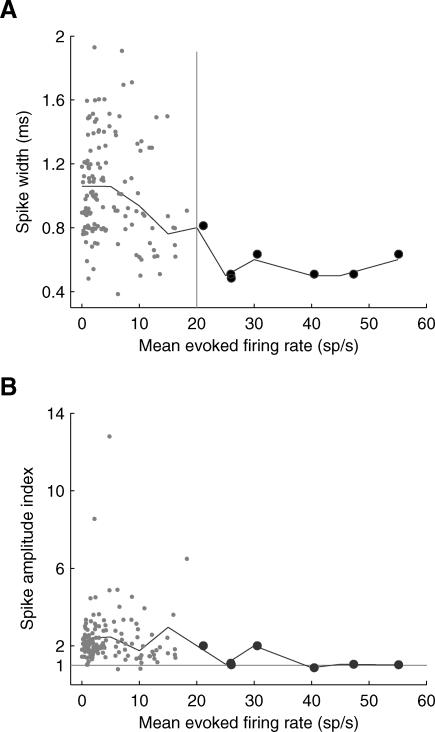
Very Responsive Neurons Might Be Narrow-Spiking Interneurons Neurons with higher evoked firing rates tended to have narrow, symmetrical spikes. (A) Spike width decreased with increasing evoked firing rate (*n* = 144 neurons). (B) Neurons with higher mean evoked firing rates displayed more symmetrical spikes. Perfectly symmetrical spikes would fall on the gray horizontal line. The large dots in both panels denote cells with mean evoked firing rates >20 sp/s. The black lines in both panels indicate average values in 5 sp/s bins. The mean evoked firing rate for each cell was computed as the mean firing rate across all response bins. In (A) all points were jittered slightly so that overlying points could be seen. One cell had a very large spike amplitude index of 34.3 (spike width 1.9 ms, mean evoked firing rate 1.3 sp/s) and was excluded from both panels. Note that due to lowpass filtering of the traces recorded with glass electrodes, spike widths may appear wider than when recorded using conventional metal extracellular electrodes.

Neurons with higher evoked firing rates tended to have narrower spikes (Figure 8A), suggesting that interneurons were overrepresented among the most responsive neurons. Indeed, the seven most responsive neurons—those with a mean evoked firing rate (computed across all octave bins and response epochs) higher than 20 sp/s—had narrow spikes with spike widths less than or equal to 0.9 ms. Neurons with high firing rates also tended to have symmetrical spikes (Figure 8B). Although spike width and shape are only at best crude surrogates for cell type, the striking correlation between these quantities and tone responsiveness suggest that a substantial fraction of the most responsive neurons may be interneurons.

## Discussion

We used cell-attached recording techniques in the auditory cortex of unanesthetized rats to measure the responses of individual neurons to a variety of acoustic stimuli; we then used this dataset to infer the stimulus-evoked activity across the population. The distribution of firing rates across the population was lognormal rather than exponential, and stimuli typically elicited a high firing rate in only about 5% of the population. Such sparse representations may offer computational advantages, including faster and more complete learning of auditory patterns.

### Low Firing Rates in Unanesthetized Auditory Cortex

Cell-attached recording differs from conventional extracellular recording methods—especially from tungsten recordings with single electrode—in its selection bias (see also [10]). In conventional recording, single-unit isolation requires neural activity and neurons with low firing rates—spontaneous or evoked—tend to be undersampled. During patch clamp recording, by contrast, the formation of an electrical seal does not require neuronal activity and the tip of a glass patch pipette is in physical contact with the neuron, so even neurons with very low firing rates are as likely to be included in the sample as those with high firing rates.

Although lognormal distributions have been used widely to describe the interspike interval distributions from a single neuron, population responses have usually reported to be exponentially distributed [6,12,25,26]. The exponential and lognormal distributions differ most dramatically at the low end: a lognormally distributed population has fewer nearly silent neurons (e.g., neurons with a firing rate lower than 0.1 sp/s) than an exponential population. However, because the cell-attached recording method that we used is not biased away from such nearly silent neurons, we could be confident that their underrepresentation in the population was not due to experimental undersampling. It would be of interest to see whether a lognormal distribution of firing rates is seen in neuronal datasets obtained using different recording techniques with similar recording biases, such as optical [36], tetrodes [37], or silicone probes [35].

Interestingly, lognormal distributions have recently been reported in another neurobiological context. The distribution of synaptic weights also follows lognormal distribution [38]. It is, however, unclear how these two observations are related and what mechanisms might give rise to such distributions of synaptic weights and firing rates.

### Lack of Columnar Organization of Response Dynamics

The rodent auditory cortex is highly organized, consisting of several auditory fields [39,40]. In several areas, including the area in which the present experiments were conducted (area A1, the primary auditory cortex), responses are organized tonotopically, meaning that neurons in a particular region tend to be tuned to similar frequencies [22]. Tonotopy represents the coarsest level of organization within an area, analogous to retinotopy in the primary visual cortex [41]. However, in the visual cortex of cats and primates, as well as some other cortical areas, neurons are further organized into columns, implying that neurons recorded in a single electrode penetration have similar response properties [42].

In our experiments, however, we failed to detect any organization beyond tonotopy. For example, nearly silent neurons could be situated very nearby responsive neurons. Thus we did not find a columnar organization of response dynamics.

Although our failure to find columnar organization is not definitive evidence that no such organization exists—absence of evidence is not evidence of absence—it is consistent with several indirect lines of evidence suggesting that columnar organization in the rodent auditory cortex may be weak. First, recent studies suggest that neurons in the visual cortex of rodents, unlike those in cats and primates, may not be organized into columns [36,43]; by analogy, it may be the auditory cortex of the rodent also lacks columnar organization. Second, in vitro experiments with acute rodent cortical slices suggest that local columnar connections may be weaker in auditory cortex than in the barrel cortex [44]. Thus it may be the functional microcircuitry of rodent auditory cortex is organized in a more subtle fashion [45].

### Highly Responsive Narrow-Spiking Neurons

We correlated neuronal responsiveness with cell type based on electrophysiological criteria. We computed spike width and spike amplitude index (as a measure of symmetry of spike waveform amplitude), expecting fast-spiking cells (likely GABAergic interneurons [33,46]) to have narrow and symmetrical spikes due to the fast repolarization [33,46,47]. Multiple features of spike waveforms seem to be required to classify a given cell as a pyramidal cell or an interneuron [35,48], with narrow-spiking cells usually considered to be interneurons. However, the presence of pyramidal cells with narrow spikes [49], and the overall complexity of various physiological and morphological features of interneurons [31,32,50], further complicate electrophysiological identification of interneurons.

Although definitive identification of interneurons requires other techniques such as morphological reconstruction, it is likely that majority of highly responsive cells in our sample were not excitatory pyramidal neurons. We speculate that the high responsiveness of inhibitory interneurons might contribute to population sparseness of stimulus-evoked responses by simply inhibiting responses of pyramidal neurons in the auditory cortex. Such inhibition could then lead to sparse communication between the primary auditory cortex and higher sensory cortical areas in awake animals.

### Sparse Representations and Optimal Stimuli

The sparse and heterogeneous responses we report are consistent with many previous single-unit studies of auditory cortex in unanesthetized animals, including the classical studies [21]; see also [51–55].

In many anesthetized preparations (e.g., barbiturate and ketamine), sound-evoked responses are typically transient [17,39,56,57]. With the resurgence of work in the awake preparation in the last decade, many researchers have emphasized the much richer repertoire of responses in the auditory cortex of awake animals, including especially sustained responses to sounds [25,58–60]. We propose that response heterogeneity is a hallmark of awake auditory cortex.

Our study complements recent work aimed at identifying “optimal” stimuli—stimuli that elicit high sustained firing rate [12,13,61]. The fact that a stimulus can be optimized to drive a particular neuron well tells us little about how this stimulus is represented across the population. Our data suggest that only a minority of neurons are engaged in the representation of many stimuli; indeed, the fact that most stimuli drive most neurons only weakly explains why finding the optimal stimulus for any given neuron can be such a challenge. Thus, although there may be an optimal stimulus for any given neuron, most stimuli are not optimal for most neurons, and so are represented sparsely across the population.

### Sparse Representations in the Cortex

The population sparseness in the awake auditory cortex we described arose through a combination of three factors. First, half of neurons failed to respond to any tone we presented. Second, responses were often brief. Third, the amplitude of responses was usually low. Thus, even though the frequency tuning of single neurons is usually broad [24], only a small fraction of neurons responded vigorously and most neurons were silent.

Experimental evidence for sparse coding has been found in a range of experimental preparations, including the visual [5,6], motor [11], barrel [10], and olfactory systems [7,62,63], the zebra finch auditory system [8], and cat lateral geniculate nucleus [9]. However, the sparseness of representations in the auditory cortex has not been explicitly addressed in previous work. Our results constitute the first direct evidence that the representation of sounds in the auditory cortex of unanesthetized animals is sparse.

Our data support the “efficient coding hypothesis,” [64] according to which the goal of sensory processing is to construct an efficient representation of the sensory environment. Sparse codes can provide efficient representations for natural scenes [2,65]. Sparse representations may also offer energy efficient coding, where fewer spikes are required compared to dense representations [66–68].

A growing body of theoretical work on sparse representations suggest they may be useful for computation [2–4,65,69,70]. Sparse representations have become increasingly important in statistical machine learning [71]. One intuition underlying this approach is that it can be easier to recognize a sparse pattern in a high-dimensional space than a dense pattern in a low dimensional space. This is illustrated in Text S1 and Figure S1, where spike trains drawn from a sparse distribution could more easily be discriminated than those drawn from a dense distribution. This discriminability in turn can make the patterns easier to learn rapidly (see Text S2 and Figure S2). Thus, an advantage of sparse cortical representations may be to facilitate rapid learning of arbitrary auditory patterns.

## Materials and Methods

### Surgery.

Sprague Dawley rats (21–30 days old) were anesthetized in strict accordance with the National Institutes of Health guidelines, as approved by the Cold Spring Harbor Laboratory Animal Care and Use Committee. A small craniotomy (maximum size of 1.5 × 1.5 mm) and durotomy were performed over the left (primary) auditory cortex. The position of the craniotomy was determined by its distance from bregma (4.5 mm posterior and 4 mm lateral), and its relationship to other bone sutures. The presence of clear auditory single-unit responses and/or local field potentials was further used as physiological criteria to confirm the location of the auditory cortex. Based on the anatomical landmarks and physiological criteria we expect that the neurons recorded in this study were in the primary auditory cortex [39].

The whole area was protected by a plastic well with removable cap. The brain surface was covered with Kwik-Cast (World Precision Instruments) between the recording sessions. An aluminum headpost was attached to the skull with Relyx Luting Cement (3M ESPE). A silver chloride ground wire was implanted subcutaneously on the back of the animal.

The animals were allowed at least 24 h of recovery before the first recording session. During the recording session, the head of the animal was fixed in the headpost holder and the animal was positioned inside a plastic tube, which provided a loose restraint for body movements. The plastic cap and Kwik-Cast were removed and the cortex covered with physiological buffer (in mM: NaCl, 127; Na_2_CO_3_, 25; NaH_2_PO_4_, 1.25; KCl, 2.5; MgCl_2_, 1; and glucose, 25) mixed with 1.5% agar. The animals sat quietly, occasionally moved their limbs, groomed, whisked, etc. The behavioral state of the animal was monitored by a closed video circuit. Excessive movement, signs of stress, or discomfort of the animal were used to indicate the end of the experiment. We recorded from each animal during several recording sessions (usually two or three sessions per rat). The number of recording sessions was limited by the total number of electrode penetrations. Any appearance of brain edema, or a change in cortex appearance, vasculature, etc. was a sign to discontinue recordings from the animal.

### Electrophysiology.

Cell-attached recordings were obtained using standard blind patch-clamp recording techniques; for details on this technique see also [17,72,73]. Electrodes were pulled from filamented, thin-walled, borosilicate glass (outer diameter, 1.5 mm; inner diameter, 1.17 mm; World Precision Instruments) on a vertical two-stage puller (Narishige). Internal solution contained (in mM): KCl, 10; KGluconate, 140; HEPES, 10; MgCl_2_, 2; CaCl_2_, 0.05; Mg-ATP, 4; Na_2_-GTP, 0.4; Na_2_-Phosphocreatine, 10; BAPTA, 10; and biocytin, 1 %, (pH 7.25); diluted to 290 mOsm. Resistance to bath was 3.5–5.0 MΩ before seal formation. Recordings were obtained using Axopatch 200B (Axon Instruments) with 2 kHz lowpass filter and a custom data acquisition system written in MATLAB (Mathworks), with a sampling rate of either 4 kHz or 10 kHz. Because cell-attached recording requires a minimum seal of only about 10–20 MΩ (compared with the >1 GΩ for whole cell recording), almost every neuron encountered can be patched.

We recorded from 166 neurons (in 25 animals), out of which we identified 145 neurons (in 24 animals) with at least eight trials per octave bin (see also section titled Cell counts, below). The search for neurons was conducted solely based on pipette's resistance and not on spiking activity. For inclusion in our sample, each cell had to generate at least one action potential (to guarantee that it was not, e.g., a glial cell). A great care was taken to exclude neurons that might have been damaged by direct contact between the pipette tip and cellular membrane. Recordings during which we observed gradual increase in spontaneous firing rate were excluded. In the rare cases, in which the spontaneous rate increased suddenly, or the electrode “broke in” after a sudden movement of the animal, we analyzed only the first stationary epoch of the recording.

Neurons were recorded from all depths (Figure 7B). The neuron appearing in Figure 2A appeared previously in a review article (Figure 2B of [1]), as did the neuron presented in Figure 2C (Figure 3 of [1]).

### Stimuli.

All experiments were conducted in a double-walled sound booth (Industrial Acoustics Company). Free-field stimuli were presented at 97.656 kHz using TDT System 3 (Tucker-Davis Technologies) connected to an amplifier (Stax SRM 313, STAX Limited), which drove a calibrated electrostatic speaker (taken from the left side of a pair of Stax SR303 headphones) located 8 cm lateral to, and facing, the contralateral (right) ear.

The main sets of stimuli consisted of 100-ms long pure-tone pips of 16, 20, or 64 different frequencies logarithmically spaced between 1–40 kHz (81% of recordings, 134 out of 166) presented at either 20, 50, 80 dB SPL (*n* = 43), or at 0, 30, 60 dB SPL (*n* = 15), or at 0, 20, 40, 60 dB SPL (*n* = 76). For the rest of recordings (19%, 32 out of 166) the stimulus protocol contained 100-ms long pure-tone pips of 28 frequencies logarithmically spaced between 2–48 kHz presented at 60 dB SPL. All tones were repeatedly presented in a fixed pseudo-random order at a rate of two tones per second. A full tuning curve was obtained for each neuron.

In 22 neurons (13% of recordings, 22 out of 166) we also presented frequency-modulated sweep stimuli. Sweeps covered the frequency range from 1 to 40 kHz, and both upward (from 1 to 40 kHz) and downward (from 40 to 1 kHz) going sweeps were presented at six different rates (25, 50, 75, 100, 125, 150 octaves/second) for each neuron (see Figure 2I for an example).

In 43 neurons (26% of recordings, 23 out of 166) we presented 100-ms long white-noise bursts at 80 dB SPL.

Natural sound stimuli were presented for 28 neurons. Of those, 23 neurons were also presented with pure tones (14% of recordings, 23 out of 166), and five neurons were presented only with natural sounds. The natural sound stimuli were taken from a commercially available audio compact disc, “The Diversity of Animal Sounds” (Cornell Laboratory of Ornithology), originally sampled at 44.1 kHz and resampled at 97.656 kHz for stimulus presentation [72]. The sounds chosen had no special relevance to the rats (unlike, e.g., rat pup calls), and therefore are less likely to engage specialized processing mechanisms; to the extent that these sounds are representative of the acoustic environment of humans, they are also representative for rats, which often share the same habitat as humans. Altogether, four natural sound segments were presented for each neuron, with at least four repeats of each segment per neuron. The segments included Jaguar call (track 3, seconds 2 to 11 for total duration of 10 s), *Bowhead Whale* (track 9, seconds 1 to 10, 10-s duration), *Knudsen's Frog* (track 11, seconds 1 to 10, 10-s duration, Figure 2J), and *Bearded Manakin* (track 19, seconds 0.1 to 5.1, 5-s duration). The peak amplitude of each segment was normalized to the ±10 V range of the TDT system, which corresponded to 80 dB SPL.

### Spike extraction and analysis.

Spikes recorded in cell-attached mode were extracted from raw voltage traces by applying a high-pass filter and thresholding (Figure 1A). Spike times were then assigned to the peaks of suprathreshold segments, and rounded to the nearest millisecond.

Individual spikes can assume very different shapes even in a single cell (Figure 1B). In some cases we observed bursts of spikes, during which spike amplitude sometimes decreased several-fold. For the cell shown in Figure 1B, both single spikes and bursts were sometimes evoked approximately 40 ms following tone termination. Such large changes in spike characteristics can result in a failure of spike detection in conventional extracellular tungsten recordings.

Spikes were recorded at a sampling rate of 4 kHz for 88 neurons in our sample (*n* = 166 neurons), and 10 kHz for the remainder of the population. For the analysis of spike shape (Figure 8), the spike waveforms recorded at 4 kHz were resampled to 10 kHz (using MATLAB resample function). We then computed the mean spike waveform, and defined *spike width* as the time difference between the peak (maximum amplitude) and valley (minimum amplitude following the peak) of the waveform. Because the spike waveforms are (re)sampled at 10 kHz, the spike widths are rounded to the nearest tenth of a millisecond. For each cell we also computed the *amplitude index*, the absolute value of peak-to-valley-ratio, of the mean spike waveform.

### Evoked response analysis.

Responses to stimuli were divided into 50-ms-duration time bins. In addition, tone-evoked responses were also binned in frequency space. We use the term *response bin* to refer to subdivision of a response in general, as defined below for various stimuli. When we explicitly refer to binning in time, or frequency, we use the terms *response epoch,* or *octave bin*, respectively.

### Tones.

Tone-evoked responses were divided into four 50-ms- long *response epochs* (Figure 3A). The *spontaneous* epoch was defined as the 50-ms-long period preceding stimulus onset. The *early* epoch was defined as the first 50 ms of stimulus duration, the *late* epoch as the last 50 ms of stimulus duration, and the *off* epoch as the first 50 ms after stimulus termination. In frequency space the responses were grouped into one-octave-wide bins, which resulted in four or five frequency bins (*octave bins*) per cell (depending on the stimulus protocol used, see Stimuli above).

The *spontaneous firing rate* for each cell was computed as a mean of firing rates across all trials in the spontaneous epoch for the given cell. *Evoked firing rates* were computed for each combination of response epoch and octave bin as a mean of firing rates of all trials in the specific octave-epoch combination (Figure 3A).

The distribution of firing rates across octave bins for each response epoch was fit with a lognormal distribution (Figure 3). To fit each distribution, the octave bins with zero firing rate were removed, and the mean and variance of the distribution of log-transformed firing rates were computed. The mean and variance obtained directly from data were then used as parameters for the normal distribution fit to log-transformed firing rates. The goodness-of-fit for each distribution was assessed using the Kolmogorov-Smirnov test.

The significance of stimulus-evoked changes in firing rates was evaluated with the Wilcoxon signed-rank test, i.e., a non-parametric paired, two-sided test of the hypothesis that the difference in firing rates between the matched trials in two different epochs comes from a distribution whose median is zero. For each octave and *early*, *late*, and *off* response epochs we tested on a trial-by-trial basis whether the stimulus-evoked firing rate increased or decreased significantly compared to the corresponding *spontaneous* epoch. For this test we also only considered cells with at least 20 trials per octave bin (69%, 100 cells out of 145).

For the analysis of *responsiveness of single neurons* (in Results see Population Response Is Sparse) the evaluation of significance involved 15 comparisons for most of the cells, because responses of most cells were binned to 15 response bins (five octave bins times three response epochs). Therefore, we used a significance criterion of either *p* < 0.0033 (for 15 comparisons, 0.05/15), or *p* < 0.0042 (for 12 comparisons, 0.05/12) to keep the overall significance criterion for each cell at *p* < 0.05. To be considered tone-responsive, a cell had to show a significant change in firing rate (increase or decrease) in at least one response bin.

For the *population response* analysis (in Results see Population Response Is Sparse and Figure 6) the response bins from all neurons were considered independent and their responsiveness was evaluated with the Wilcoxon signed-rank test using a significance criterion of *p* < 0.01. To evaluate the population response in the *early* response epoch (Figure 6A), the fraction of bins showing a significant increase, a significant decrease, or no change in the firing rate was computed for each octave bin in the *early* response epoch. The fraction of responsive bins in the *early* response epoch was then defined as the mean of the octave-bin fractions in the epoch. Analogous computations were carried out for the *late* and *off* response epochs. To compute the population response across all epochs the fractions of responsive bins were computed from all response bins (from all neurons) pooled together.

Careful inspection revealed no clear examples of frequency tuning sharper than about one octave, suggesting that it would be appropriate to pool together responses to tones within an octave. To confirm systematically that our results were robust to this choice we repeated this analysis with half-octave wide (i.e., narrower) frequency bins, two, and four octaves wide (i.e., wider) frequency bins, and 50-ms-long response epochs. To control for neurons with more transient or sustained responses we performed the population response analysis with 25, 75, and 100 ms duration response epochs and one-octave-wide frequency bins. The results of these analyses, however, were the same as for the basic analysis with one-octave-wide frequency bins and 50-ms-duration response epochs (unpublished analysis).

### Frequency-modulated sweeps.

Responses to frequency-modulated (FM) sweeps were subdivided to 50-ms-duration response epochs. Slower sweeps, with 25 or 50 octaves/second, contained four or two 50-ms epochs, respectively, during the stimulus presentation. Faster sweeps (75, 100, 125, 150 octaves/second) contained one 50-ms epoch. For all sweep rates we also added an *off* epoch starting either at the sweep termination (for 25, 50, 75, 100 octaves/second), or immediately after the response epoch (for 125, 150 octaves/second). Each response was thus divided into 32 response bins (including upward and downward moving sweeps). For the analysis of significance of sweep-evoked responses in individual neurons, we therefore used a significance criterion of *p* < 0.0016 (0.05/32). To compute the population response to FM-sweeps all response bins were considered statistically independent, and their responsiveness was computed using a significance criterion of *p* < 0.01.

### White-noise bursts.

Responses to 80-dB white-noise bursts were divided into four 50-ms-long response epochs (spontaneous, early, late, and off) analogous to the tone response epochs described above.

### Natural sounds.

Responses to natural sounds were also divided to 50-ms- duration response epochs. 10-s-long segments thus contained 200 response bins each, and 5-s-long segments contained 100 response bins. Natural sound-evoked responses were used only for the analysis of stimulus-evoked changes in firing rate, because none of the recordings met our criterion for the test of evoked response significance (i.e., at least 20 trials per response bin).

### Cell counts.

For the analysis of *stimulus-evoked changes in firing rate* (Table 1, Figures 4, 5, and 7C), we identified neurons with at least eight trials per response bin (five trials for natural sounds, three trials for white-noise bursts). For the analysis of *significance of stimulus-evoked responses* (Figure 6), we identified neurons with at least 20 trials per response bin.

We recorded from 166 neurons (100%), while presenting pure-tone pips. For further analysis of firing rates evoked by 50- or 60-dB tones we identified 145 neurons (87%) with at least eight trials per response bin. For the analysis of evoked response significance we further identified a subset of 100 neurons (60%) with at least 20 trials per response bin.

For 91 neurons (55%) we also presented 30- or 40-dB tones. All of these neurons were used for the firing rate analysis, and 62 neurons (37%) from this subset—those with at least 20 trials per response bin—were used for the analysis of evoked response significance. Accordingly, out of 43 neurons (26%) presented with 80-dB tones we selected 22 (13%) for firing rates analysis, and six (4%) with at least 20 trials per octave bin for the analysis of evoked response significance.

FM sweeps were presented for 22 neurons, all of which were used for the firing rates analysis. Seventeen neurons with at least 20 trials for each sweep rate and direction were further selected for the analysis of significance of sweep-evoked responses.

White-noise bursts (80 dB) were presented for 43 neurons. For the analysis of evoked firing rates we identified 23 neurons (55%) with at least three trials per response bin.

Natural sounds were presented for 28 neurons. Twenty-seven neurons with at least five trials for each natural sound segment were identified for the analysis of stimulus-evoked firing rates. Bootstrap resampling showed that the smaller sample size did not influence our estimates of fraction of well-driven response bins (see Results.)

## Supporting Information

 Figure S1 Spike Patterns Generated from Sparse Distributions of Firing Rates Are More Distinct than Patterns Generated from Dense Distributions (20 KB PDF)

Figure S2 Hebbian Learning Is Easier and Faster for Neuronal Patterns Derived from Sparse Distributions of Firing Rates (15 KB PDF)

Figure S3 Highly Responsive Bins Are Distributed Unevenly in the Population (4 KB PDF)

Figure S4 Tone-Evoked Responses in the Auditory Cortex of Unanesthetized Rats Are Heterogeneous (120 KB PDF)

Figure S5 Tone-Evoked Responses in the Auditory Cortex of Unanesthetized Rats Are Heterogeneous (134 KB PDF)

Figure S6 Tone-Evoked Responses in the Auditory Cortex of Unanesthetized Rats Are Heterogeneous (58 KB PDF)

Figure S7 Tone-Evoked Responses in the Auditory Cortex of Unanesthetized Rats Are Heterogeneous (47 KB PDF)

Figure S8 Tone-Evoked Responses in the Auditory Cortex of Unanesthetized Rats Are Heterogeneous (35 KB PDF)

Text S1 Sparse Coding for Reliable Stimulus Representation and Learning. (24 KB PDF)

Text S2 Hebbian Learning for Sparse Representations (21 KB PDF)

## Abbreviations

dB: decibel
GABA: gamma-aminobutyric acid
ISI: interspike interval
kHz: kilohertz
ms: millisecond
SPL: sound pressure level
sp/s: spikes/second
