# Supplementary material for: Sparse Representation of Sounds in the Unanesthetized Auditory Cortex
Source: PLoS Biol. 2008 Jan 29;6(1):e16. doi: 10.1371/journal.pbio.0060016 (PMC2214813; doi:10.1371/journal.pbio.0060016)
Supplement: Figure S1 — (20 KB PDF) [file pbio.0060016.sg001.pdf]

Sparse Representation of Sounds in the Unanesthetized Auditory Cortex

Tomáš Hromádka, Michael R. DeWeese and Anthony M. Zador

Figure S1

Sparse coding for reliable stimulus representation and learning

Fig. S1 caption:

Spike patterns generated from sparse distributions of firing rates are dominated by a small number of neurons with higher firing rates and are more distinct than patterns generated from dense distributions (see *Text S1* for details.)

(A), **(B)** Two examples of spike patterns generated from sparse (lognormal) distributions of firing rates. The top panels show two distributions of firing rates for 200 neurons drawn from the same underlying lognormal distribution matching our data. Each of the bottom panels show 100 spiking patterns (columns) generated for each neuron in the population, with summary neuronal activity for the 100 patterns depicted as a histogram on the right side of each panel; note the tall peaks in each histogram that greatly facilitate the discriminability between these two sparse patterns of activity. Each spike pattern represents a snapshot of neuronal activity during 10 ms (see *Text S1*.) Dots represent the occurrence of at least one spike in 10 ms wide windows.

(C), **(D)** Two examples of spike patterns generated from dense (normal) distribution of firing rates. Parameters for the normal distribution were chosen to match mean firing rate, total amount of spikes, and entropy of the lognormal distribution. Same format as in (A), and (B).

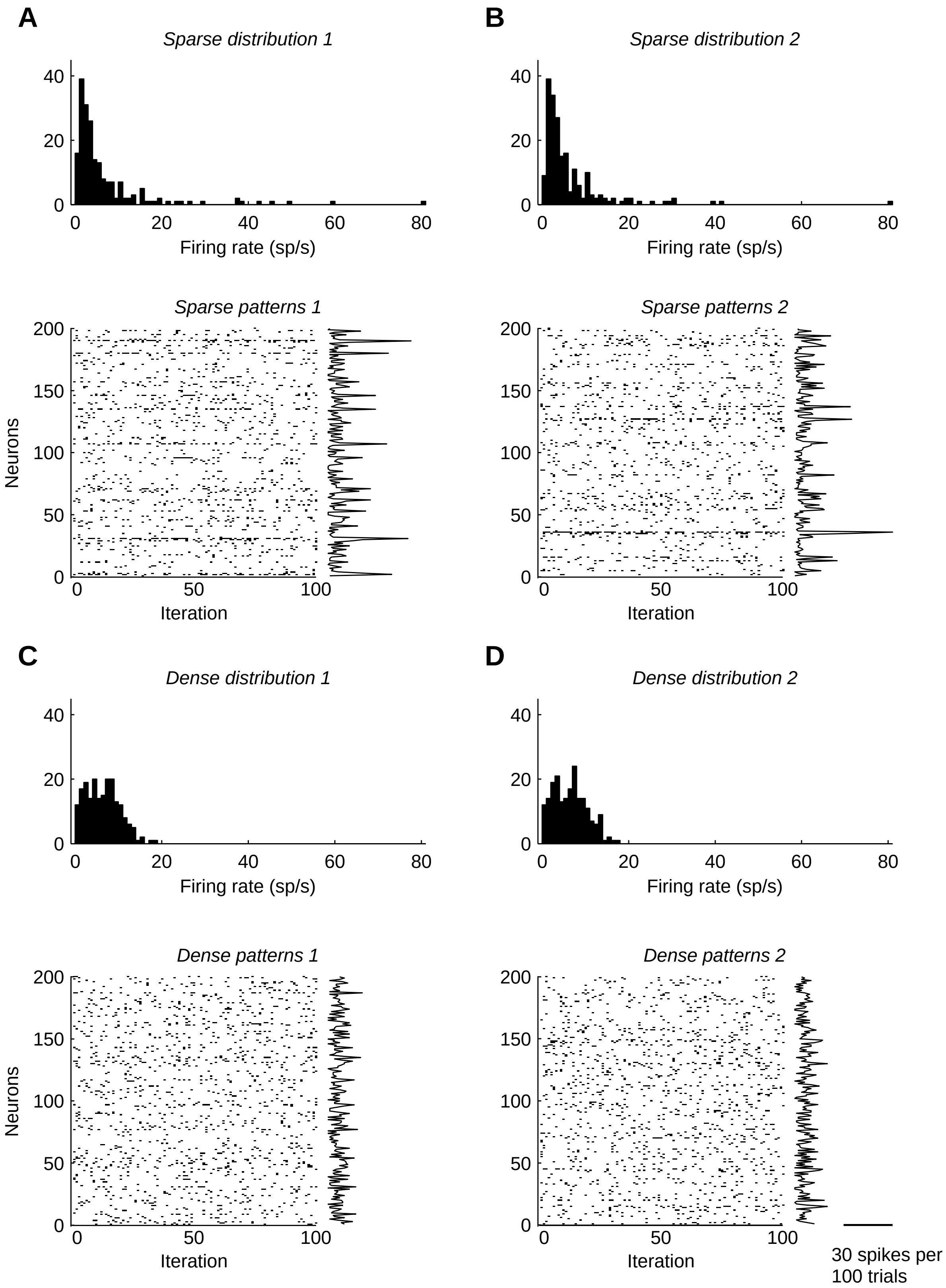
A
Sparse distribution 1
40
20
0
0
20
40
60
80
Firing rate (sp/s)
B
Sparse distribution 2
Firing rate (sp/s)
Sparse patterns 1
Neurons
200
150
100
50
Iteration
Sparse patterns 2
Iteration
C
Dense distribution 1
Firing rate (sp/s)
D
Dense distribution 2
Firing rate (sp/s)
Dense patterns 1
Neurons
Iteration
Dense patterns 2
Iteration
30 spikes per
100 trials

Figure S1:
