# Supplementary material for: Sparse Representation of Sounds in the Unanesthetized Auditory Cortex
Source: PLoS Biol. 2008 Jan 29;6(1):e16. doi: 10.1371/journal.pbio.0060016 (PMC2214813; doi:10.1371/journal.pbio.0060016)
Supplement: Figure S2 — (15 KB PDF) [file pbio.0060016.sg002.pdf]

Sparse Representation of Sounds in the Unanesthetized Auditory Cortex

Tomáš Hromádka, Michael R. DeWeese and Anthony M. Zador

Figure S2

Hebbian learning for sparse representations

Fig. S2 caption:

Hebbian learning is easier and faster for neuronal patterns derived from sparse distributions of firing rates (see *Text S2* for details.)

(A) The distribution of firing rates of dense and sparse populations used in the simulation. The parameters of the sparse (lognormal) distribution were taken from the observed data; the parameters of the dense distribution were matched in mean firing rate and entropy.

(B) Sample rasters for the target (*red*) and background (*black*) patterns, and the associated firing rate histograms. A spike pattern from the target distribution is presented to a model neuron with Hebbian synapses every other trial; non-target spike patterns presented on alternating (background) trials are drawn from a new distribution each trial. The target sparse distribution contains a few outliers that dominate learning, whereas the absence of such outliers in the dense distribution leads to slower and less efficient learning.

(C) The ratio of responses elicited by target patterns on a model neuron with Hebbian synapses (see Eq. 2 in *Text S2*) to non-target responses is shown. The ratio grows faster and asymptotes to a higher level for the sparse distribution, indicating that learning is faster and more efficient.

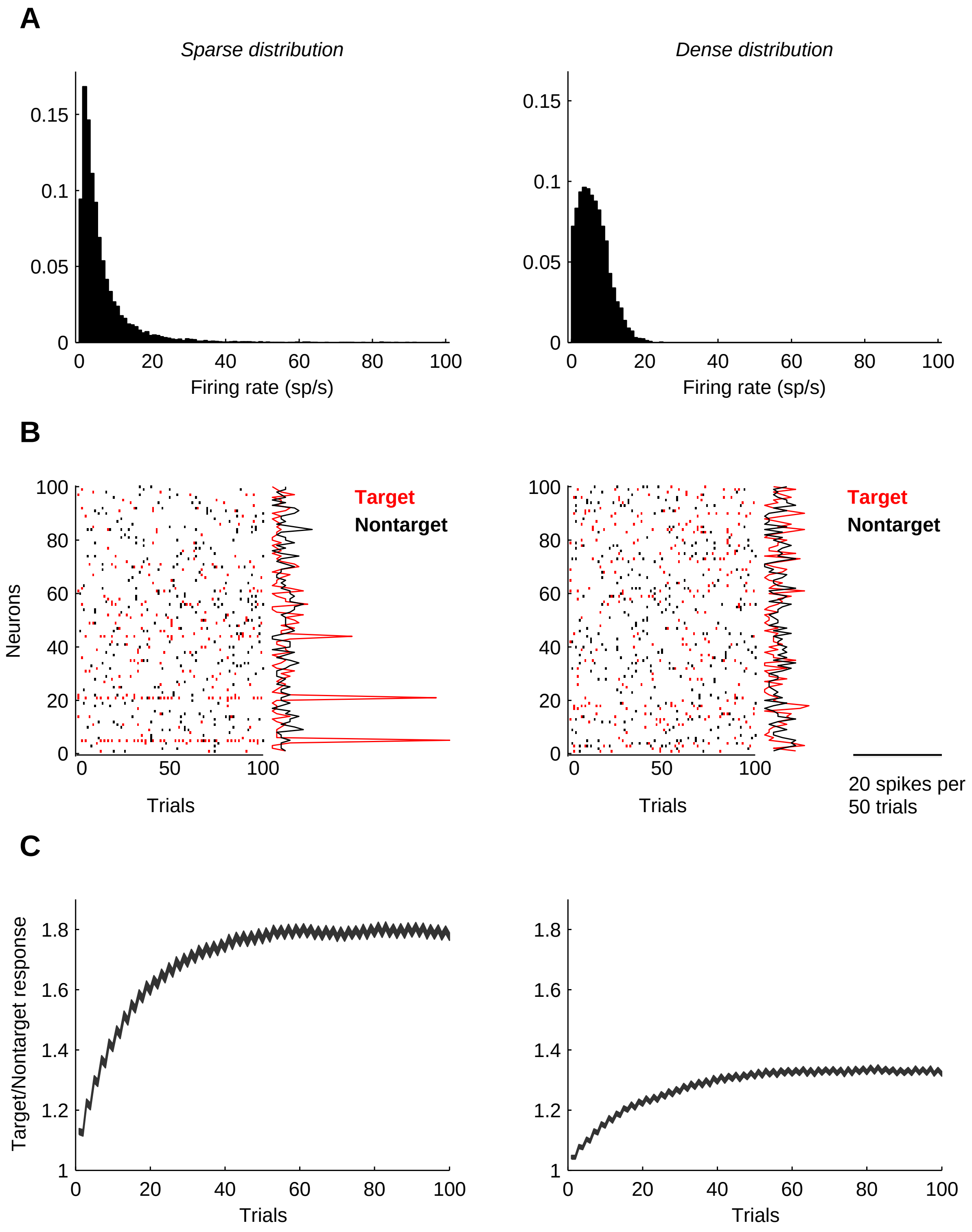
A
Sparse distribution
Dense distribution
Firing rate (sp/s)
Firing rate (sp/s)
B
Target
Nontarget
Neurons
Trials
Trials
20 spikes per
50 trials
C
Target/Nontarget response
Trials
Trials

Figure S2:
