# Supplementary material for: Sparse Representation of Sounds in the Unanesthetized Auditory Cortex
Source: PLoS Biol. 2008 Jan 29;6(1):e16. doi: 10.1371/journal.pbio.0060016 (PMC2214813; doi:10.1371/journal.pbio.0060016)
Supplement: Figure S3 — (4 KB PDF) [file pbio.0060016.sg003.pdf]

Sparse Representation of Sounds in the Unanesthetized Auditory Cortex

Tomáš Hromádka, Michael R. DeWeese and Anthony M. Zador

Figure S3

Highly responsive bins are distributed unevenly in the population

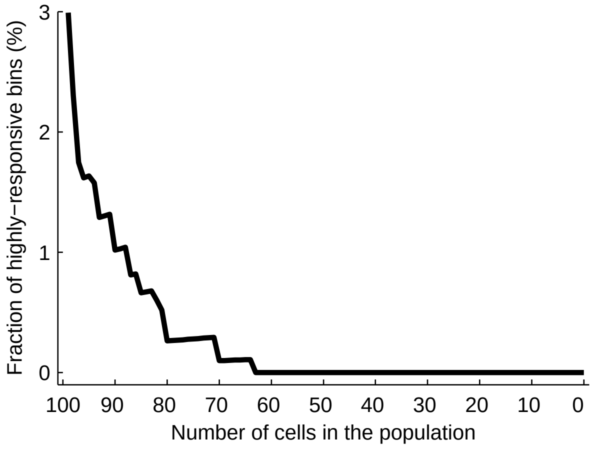

Figure S3: To evaluate how evenly the highly responsive bins are distributed in the neuronal population, we computed fractions of highly responsive bins (i.e. bins with evoked firing rate change >20 sp/s) for neuronal populations of different sizes. Cells for which we assessed response significance (n=100, see *Materials and Methods* and Fig. 6B in the main text) were sorted in descending order according to the number of significantly responsive bins. In each iteration, the cell with the highest number of significantly responsive bins was removed from the population and the fraction of highly responsive bins was recomputed. This analysis shows that only a subset of neurons are highly responsive.
