# Supplementary material for: Sparse Representation of Sounds in the Unanesthetized Auditory Cortex
Source: PLoS Biol. 2008 Jan 29;6(1):e16. doi: 10.1371/journal.pbio.0060016 (PMC2214813; doi:10.1371/journal.pbio.0060016)
Supplement: Figure S8 — (35 KB PDF) [file pbio.0060016.sg008.pdf]

Sparse Representation of Sounds in the Unanesthetized Auditory Cortex

Tomáš Hromádka, Michael R. DeWeese and Anthony M. Zador

Figure S8

Neuronal responses are heterogeneous

Figs. S4–S8 caption

Tone-evoked responses in the auditory cortex of unanesthetized rats are heterogeneous. The panels in Figs. S4–S8 show response dynamics of all neurons (n=100) for which we assessed response significance to 50–60 dB tones. In each panel, dots represent individual spikes, and the gray shaded region indicates the tone duration (100 ms).

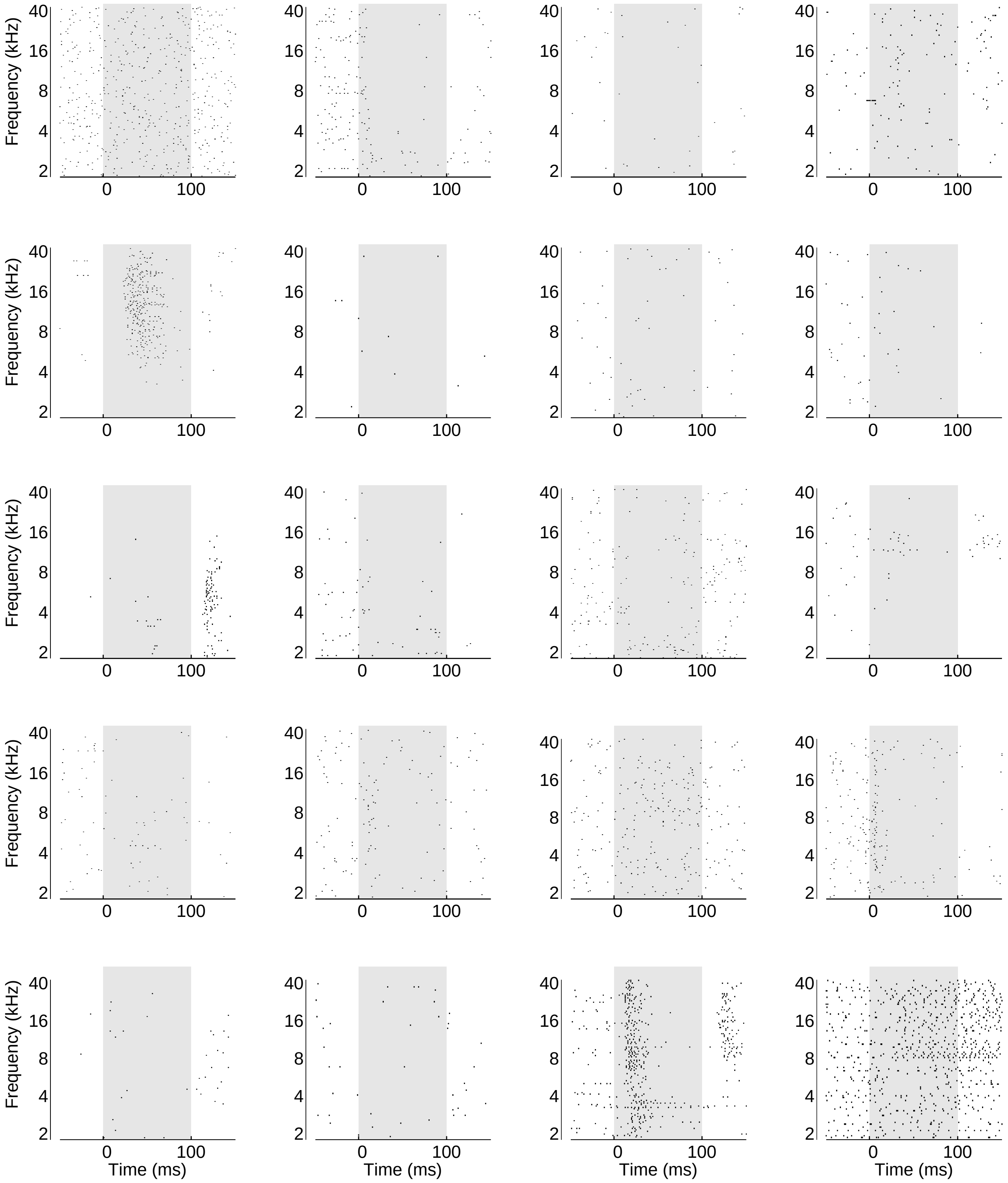

Figure S8:
