# Supplementary material for: Sparse Representation of Sounds in the Unanesthetized Auditory Cortex
Source: PLoS Biol. 2008 Jan 29;6(1):e16. doi: 10.1371/journal.pbio.0060016 (PMC2214813; doi:10.1371/journal.pbio.0060016)
Supplement: Text S1 — (24 KB PDF) [file pbio.0060016.sd001.pdf]

Sparse Representation of Sounds in the Unanesthetized Auditory Cortex

Tomáš Hromádka, Michael R. DeWeese and Anthony M. Zador

Text S1

Sparse coding for reliable stimulus representation and learning

Why should the cortical representation of an acoustic stimulus be sparse? Several explanations have been advanced. One set of proposals focuses on the energy used by neural activity; sparse representations involve fewer spikes and thereby minimize the energetic costs associated with a neural representation [1–3]. Other proposals focus on the advantages of sparse representations for computation. For example, it has been suggested that the statistics of natural sensory environments are sparse, and that a sparse code provides a natural match to such environments [4–6]. Recently, it was shown how a sparse overcomplete representation could be used to solve the "cocktail party problem" (i.e. separate a single auditory stream from several mixed together) [7].

Fig. S1 demonstrates one benefit of sparse representations in the present context. This simple example is not intended as a model of auditory cortex, but merely to illustrate some of the basic intuitions underlying sparse representations. We compare the representation of auditory stimuli by two hypothetical neuronal populations, one dense and the other sparse. In the sparse population (Fig. S1AB), firing rates are drawn from the lognormal distribution we observed, whereas in the dense population firing rates are drawn from a hypothetical Gaussian distribution (Fig. S1CD), the mean firing rate and entropy (a measure of the representational capacity) of which were matched to the observed distribution (see *Supporting methods* below).

To examine the ability of each population to represent a pair of distinct stimuli, we draw two patterns of firing rates (P1 and P2), corresponding to the two stimuli, from the sparse distribution; and similarly draw two patterns from the dense distribution. It seems reasonable to suppose that a good neural representation of a pair of distinct stimuli should allow the stimuli to be easily discriminated. Specifically, since at any instant an organism only has access to a set of spikes rather than to the underlying firing rates, the stimuli should be discriminated on the basis of the pattern of spikes across the population—that is, on the basis of the spikes representing a single instantiation of the firing rates. The question, then, is how well a spike train drawn from P1 can be discriminated from a spike train drawn from P2, and how this discriminability depends on whether P1 and P2 are drawn from sparse or dense distributions.

The spike trains drawn from the sparse distribution are dominated by a few outliers—a few neurons with high firing rates—which can be used to reliably discriminate the pattern P1 from P2 even by eye. By contrast, the absence of such outliers in the spike trains drawn from the dense distribution makes it difficult to discriminate these patterns. This intuition can be quantified by a discriminability measure Q (see *Supporting methods* below), which confirms that the sparse representations were consistently more easily discriminated than the dense ones (Q=5.0 for sparse representations, compared to Q=1.9 for dense representations). Moreover, model neurons with Hebbian synapses learned to discriminate sparse patterns more rapidly and completely than dense patterns (see *Text S2*). Thus the presence of a handful of neurons with high firing rates can facilitate stimulus discrimination and learning in a simple model.

Supporting methods for simulation experiments

To compare stimulus representations in sparse and dense neuronal populations we computed the similarity of pairs of spike patterns that were either both generated by neurons with firing rates drawn from lognormal (sparse) distributions, or both

generated by neurons with firing rates drawn from truncated Gaussian (dense) distributions. For the simulated lognormal distribution of firing rates we used parameters given by the distribution of spontaneous firing rates (Fig. 3BC), with a mean of 1.3 sp/s, and a standard deviation of 1.0 sp/s, both on a logarithmic scale. To create a corresponding (truncated) Gaussian distribution of firing rates we matched the mean firing rate and entropy of the lognormal distribution, which corresponded to a Gaussian with a mean of 4.2 sp/s and a standard deviation of 5.2 sp/s, on a linear scale, with negative firing rates replaced by rates drawn again from the same distribution until the distribution contained only non-negative firing rates.

To simulate responses of neuronal populations, we first drew two patterns, X and Y, of firing rates—each of these rate patterns was a vector of n=200 values, representing the firing rate of each neuron in the population. For the sparse patterns each element of each vector was drawn from the sparse distribution; similarly for the dense patterns, each element was drawn from the dense distribution. We then generated 100 individual spike patterns from each rate pattern by treating each element as the rate (in a 10 ms window) for a Poisson process.

We defined the discriminability $q(X,Y)$ between a pair of rate patterns (i.e. between two sets of firing rates X and Y over a population of neurons) as:

$$q(X,Y) = \frac{\langle x \cdot x' \rangle \langle y \cdot y' \rangle}{\langle x \cdot y \rangle^2} \qquad (1)$$

where x and x' are patterns of spike counts drawn from X, and y and y' are spike patterns drawn from Y; the brackets denote averages over all pairs of spike patterns (instantiations of the Poisson spike trains). We then used the average of this quantity over pairs of rate patterns to quantify how different spike patterns drawn from the different underlying distributions were:

$$Q = \langle q(X,Y) \rangle_{rates} \qquad (2)$$

We thus could compare Q for patterns X and Y drawn from a sparse distribution with Q for patterns X and Y drawn from a dense distribution.

The higher the discriminability score, the more discriminable are spike patterns drawn from one pattern of rates when compared to another pattern of rates. A discriminability score close to 1 means that spike patterns from one rate pattern are (on average) the same as spike patterns from another rate pattern.

References

[1] Levy WB, Baxter RA (1996) Energy efficient neural codes. Neural Comput 8:531–543.

[2] Attwell D, Laughlin SB (2001) An energy budget for signaling in the grey matter of the brain. J Cereb Blood Flow Metab 21:1133–1145.

[3] Laughlin SB, Sejnowski TJ (2003) Communication in neuronal networks. Science 301:1870–1874.

[4] Olshausen BA, Field DJ (1997) Sparse coding with an overcomplete basis set: a strategy employed by V1? Vision Res 37:3311–3325.

[5] Lewicki MS (2002) Efficient coding of natural sounds. Nat Neurosci 5:356–363.

[6] Olshausen BA, Field DJ (2004) Sparse coding of sensory inputs. Curr Opin Neurobiol 14:481–487.

[7] Asari H, Pearlmutter BA, Zador AM (2006) Sparse representations for the cocktail party problem. J Neurosci 26:7477–7490.
