# Supplementary material for: Sparse Representation of Sounds in the Unanesthetized Auditory Cortex
Source: PLoS Biol. 2008 Jan 29;6(1):e16. doi: 10.1371/journal.pbio.0060016 (PMC2214813; doi:10.1371/journal.pbio.0060016)
Supplement: Text S2 — (21 KB PDF) [file pbio.0060016.sd002.pdf]

Sparse Representation of Sounds in the Unanesthetized Auditory Cortex

Tomáš Hromádka, Michael R. DeWeese and Anthony M. Zador

Text S2

Hebbian learning for sparse representations

We have studied a single neuron model with Hebbian synapses to compare learning of neuronal activity patterns arising in sparse (lognormal) or dense (Gaussian) distribution of firing rates (Fig. S2). Parameters for sparse and dense distributions were estimated from data, as described in *Text S1*. Briefly, the sparse (lognormal) distribution of firing rates had mean=1.3 sp/s, and std=1.0 sp/s (both on a logarithmic scale). Parameters of the dense (Gaussian) distribution (mean=4.2 sp/s, std=5.2 sp/s) of firing rates were chosen so that the two distributions had the same mean firing rate and entropy. Elements from the dense distribution with negative firing rates were discarded and drawn again from the same distribution until the dense distribution contained only non-negative firing rates.

From the firing rate distributions we then generated *training set* of k firing rate patterns, each consisting of n neurons with firing rates chosen randomly from the same firing rate distribution. One neuronal pattern was randomly chosen to be the *target* pattern, and the rest were labeled as *nontarget* patterns. Every other pattern in the training set was then replaced by the target pattern. From the training set of firing rate patterns we then generated a set of spike patterns representing population outputs during 10 ms windows: each firing rate was replaced by a number of spikes generated by a Poisson process (λ=firing rate) in a 10 ms window. Each input spike pattern in the training data set thus represented a 10 ms snapshot of neuronal spiking activity. Note that while the target *firing rate patterns* were identical, the actual target *spike patterns* were not (although they were similar, see *Text S1*), because they were generated by a stochastic Poisson process.

We simulated learning in a single sigmoidal neuron with n inputs (corresponding to n neurons in the input patterns) and one output. In each trial (a single presentation of input pattern) the sigmoidal neuron computed its output (response) as a weighted sum of its inputs transformed by a sigmoid function. After the trial, the neuronal response was used to compute a new set of synaptic weights.

Thus, the neuronal response y^t in trial t was computed as:

$$y^t = \sigma\left(\sum_{i=1}^{n} w_i^{t-1} x_i^t\right), \tag{1}$$

where w_i^{t-1} are current synaptic weights (computed in the previous trial) associated with current inputs x_i^t, and $\sigma = 1/(1 + e^{-100z+6.2})$ is a sigmoid function with z as parameter (i.e. $z = \sum_i w_i x_i$).

The weights w_i were initialized with values from 0 to 1 drawn from a uniform distribution, and then normalized so that $\sum w_i = 1$. After presentation of each input pattern, weights were adapted according to a Hebbian learning rule:

$$w_i^t = w_i^{t-1} + \eta\, y^t x_i^t \tag{2}$$

where $t = 1, \ldots, k$ denotes trials (presentations of individual input patterns), η is the learning rate, y^t is the current neuronal response (i.e. postsynaptic activity), x_i^t denotes the ith input in the current trial (i.e. presynaptic activity), w_i^0 is the set of initial synaptic weights. w_i^k is thus the final set of synaptic weights after presenting all k input patterns.

Fig. S2 documents the learning process in detail for both sparse and dense representations. Panel A shows the two distributions of firing rates from which we generated input firing rate patterns.

Panel B shows examples of input spike patterns generated from firing rate patterns for n=100 neurons and k=100 trials. Red dots show spikes generated by the target pattern (every second trial), black dots show spikes from the nontarget patterns.

The histograms on the right side of each of the input patterns show spiking activity for target patterns (*red* line), and nontarget patterns (*black* line). Note that the red histogram is easily distinguishable from the black histogram for the sparse distribution.

We repeated the simulation experiment 1000 times, each time drawing a different set of n=100 neurons, and k=100 trials. Panel C shows the ratio of target to nontarget responses averaged across experiments (line thickness represents the standard error of the mean). The value at each point (trial t) was computed as $\sigma(\sum_i w_i^t x_i^t)$ for each experiment and then averaged across experiments.
